# Nanowire implosion under laser amplified spontaneous emission pedestal irradiation

**DOI:** 10.1038/s41598-023-48090-9

**Published:** 2023-11-24

**Authors:** J. F. Ong, A. Zubarev, A. C. Berceanu, M. Cuzminschi, O. Tesileanu

**Affiliations:** 1 Extreme Light Infrastructure - Nuclear Physics (ELI-NP), Horia Hulubei National Institute for R &D in Physics and Nuclear Engineering (IFIN-HH), 30 Reactorului Street, 077125 Bucharest-Măgurele, Romania; 2 National Institute for Laser, Plasma and Radiation Physics, 077125 Bucharest-Măgurele, Romania; 3 Horia Hulubei National Institute for R &D in Physics and Nuclear Engineering (IFIN-HH), 30 Reactorului Street, 077125 Bucharest-Măgurele, Romania; 4 Faculty of Physics, University of Bucharest, 077125 Bucharest-Măgurele, Romania

**Keywords:** Plasma physics, Laser-produced plasmas

## Abstract

Nanowire array targets exhibit high optical absorption when interacting with short, intense laser pulses. This leads to an increased yield in the production of accelerated particles for a variety of applications. However, these interactions are sensitive to the laser prepulse and could be significantly affected. Here, we show that an array of aligned nanowires is imploded when irradiated by an Amplified Spontaneous Emission pedestal of a $1\,\text{PW}$ laser with an intensity on the order of $10^{11}\, \mathrm {W \, cm^{-2}}$. Using radiation hydrodynamics simulations, we demonstrate that the electron density profile is radially compressed at the tip by the rocket-like propulsion of the ablated plasma. The mass density compression increases up to $2.9\times$ when a more dense nanowire array is used. This is due to the ablation pressure from the neighboring nanowires. These findings offer valuable information for selecting an appropriate target design for experiments aimed at enhancing production of accelerated particles.

## Introduction

Nanostructured targets allow deep laser penetration when interacting with short intense laser pulses. Different heating mechanisms such as vacuum heating^1^ and $J \times B$ heating^2^ occur simultaneously. This results in more than 70% laser energy absorption^3^ and leads to enhanced proton acceleration^4–6^, high pressure generation^7,8^, microscale nuclear fusion^9^, high brilliance gamma ray and x ray production^10,11^. The unique features of the laser-nanowire interaction are sensitive to the laser prepulse and the structure could be significantly affected.

The laser pulse of a modern PW-class laser, with a peak intensity of up to $10^{23} \, \mathrm {W \, cm^{-2}}$, is usually preceded by a prepulse. The prepulse consists of nanoseconds Amplified Spontaneous Emission (ASE) pedestal, picoseconds exponential ramp, and sub-picoseconds pulses with intensities ranging from $10^9 - 10^{14} \, \mathrm {W \, cm^{-2}}$^12,13^. A long prepulse can impact laser-driven experiments in a number of ways, including the creation of high harmonics from plasma guiding^14^, a decrease in laser energy conversion caused by critical density pre-plasma^15^, and the formation of plasma instabilities and turbulence^16,17^.

These problems are often mitigated by improving the laser prepulse contrast^18–21^. Several methods exist for improving the laser contrast, including Nonlinear Elliptical Polarization Rotation (NER)^22,23^, Double Chirped Pulse Amplification^24^, and Cross-Polarized Wave (XPW) generation^25–27^, Plasma Mirrors (PM)^28–30^, Optical Parametric Chirped Pulse Amplification (OPCPA)^12^, relativistic guiding^31^ and saturable absorbers^32^. An ultra-high contrast ($10^{14}-10^{19}$) is possible with the combination of OPCPA, Cross-Polarized Wave (XPW), and PM^12^.

The contrast between levels of $10^{13}-10^{11}$ for TW-class lasers does not significantly affect the interaction with structured targets because the intensity of the ASE pedestal is below the threshold for laser-induced breakdown (LIB) for most metallic target i.e. $\lesssim 10^{9} \, \mathrm {W \, cm^{-2}}$^33^. A higher peak laser powers such as $1\, \text{PW}$ and $10\, \text{PW}$ laser at ELI-NP^34–36^ are of concern, as the intensity of the ASE pedestal may reach the order of $10^{11} \, \mathrm {W \, cm^{-2}}$. In addition, the surface morphology of the target would reduce the melting threshold by a factor of 3 to 5 compared to flat targets^37^. This could potentially lead to the destruction of the nanostructures before the arrival of the main pulse. To date, there has been a lack of detailed studies of the effects of the laser pre-pulse on nanostructured targets.

In this article, we present the results of the numerical simulation depicting the implosion of a nanowire when irradiated by a $1 \, \text{PW}$ laser having an ASE pedestal contrast reaching $10^{11} \, \mathrm {W \, cm^{-2}}$ at time $-250 \, \text{ps}$ before the main peak intensity. The nanowire tip is rapidly heated and leading to plasma ablation within the first $100 \, \text{ps}$. The nanowire is then compressed by the rocket-like propulsion of the ablated plasma. The nanowire is imploded by the laser ASE pedestal. The electron and mass density are then compressed. Smaller nanowire spacing in an array lead to more pronounced mass compression due to ablation pressure from adjacent nanowires. This results in a mass compression of $2.9\times$ for nanowires located at the center of the laser spot when irradiated with $500\, \text{ps}$ ASE pedestal. This research facilitates the selection of the appropriate target design for experiments aimed at improving production of accelerated particles.

## Result

### Single nanowire

**Figure 1:**
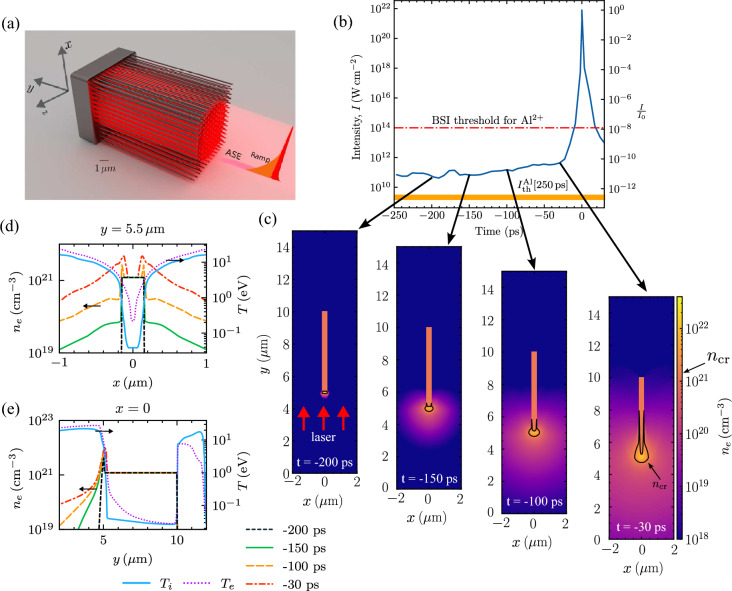
flash simulation of a single nanowire irradiated by the ASE of a $1 \, \text{PW}$ laser pulse. (**a**) The illustration of irradiation configuration and demonstration of each section of the laser prepulse. (**b**) The measured temporal contrast of the $1 \, \text{PW}$ laser pulse at ELI-NP^38,39^. The simulation is performed up to $t=-10\,\text{ps}$ before the onset of strong-field ionization for Al$^{2+}$, which corresponds to $240\,\text{ps}$ of ASE irradiation. The target is at least once ionized by collisional ionization during the ASE irradiation (See the Ionization in the “Methods” Section). The intensity of the ASE is $\sim 10^{11} \, \mathrm {W \, cm^{-2}}$. The yellow band indicates the LIB threshold, $I_{{{\text{th}}}}^{{{\text{Al}}}}$ of aluminum for a pulse duration of $250\,\text{ps}$. The main pulse has a maximum intensity of $I_0\sim 10^{22}\, \mathrm {W \, cm^{-2}}$ at $t=0$. (**c**) The time evolution of electron number density at $t=-200, \,-150,\,-100$  and $-30 \, \text{ps}$ of the irradiation with laser prepulse. The laser is irradiated at normal incidence on the nanowire in the $+y-$direction. The black solid line contours indicate the critical plasma density, $n_\text{cr}=1.64\times 10^{21} \, \mathrm {cm^{-3}}$. (**d**, **e**) Transverse and longitudinal electron number density profile at different moments in time along $y=5.5 \, \mathrm {\mu m}$ and $x=0$, respectively. The electron and ion temperature after the ASE heating at $t=-30\,\text{ps}$ are plotted.

We start the simulations with a simple configuration by considering a single aluminum nanowire. The simulations were performed by using the two-dimensional (2D) Radiation-Hydrodynamics (RHD) code flash-4.6.2^40^, which is a finite-volume Eulerian code operating on block-based Adaptive Mesh Refinement (AMR). The laser with $1\,\text{PW}$ peak power and wavelength $\lambda _\text{L}=820 \, \text{nm}$ at ELI-NP is irradiated on the nanowire target at normal incidence as illustrated in Fig. 1a. Each section of the laser prepulse is also demonstrated. The intensity of the laser ASE pedestal after focused into $R_x=2\,\mathrm {\mu m}$ spot radius is $\sim 10^{11} \, \mathrm {W \, cm^{-2}}$, which is above the LIB threshold $I^\text{Al}_\text{th}\sim 2\times 10^{9} \, \mathrm {W \, cm^{-2}}$ of aluminum for the pulse duration of $250 \, \text{ps}$^33,41^ as depicted in Fig. 1b. An aluminum nanowire of $300\,\text{nm}$ diameter and $5\,\mathrm {\mu m}$ length is place at the center of $4 \times 15\,\mathrm {\mu m}^2$ simulation box with fully ionized electron density $n_e=7.83 \times 10^{23}\,\mathrm {cm^{-3}}$. Details of the simulation is described in the Section “Methods”.

Figure 1c shows the evolution of the electron number density of the aluminium nanowire at different times. The ablation starts at the tip of the nanowire at $t=-200\, \text{ps}$. The ablation at the tip continues at $t=-150\, \text{ps}$, while the rest of the nanowire that is not heated by the laser retains its shape. The intensity of the ASE pedestal gradually increases by half at $t=-100\, \text{ps}$. The lateral profile of the electron density begin to shrink. The electron number density at the tip surpasses the critical density, $n_\text{cr}=m\omega _\text{L}^2/4\pi e^2$, as annotated by the line contour. The critical density, $n_\text{cr}=1.64\times 10^{21} \, \mathrm {W\,cm^{-2}}$ for $\lambda _\text{L}=820 \, \text{nm}$. The critical density contour expands to more than half the length of the nanowire at $t=-30\, \text{ps}$. Plasma beyond the critical density contour is opaque to the laser ASE pedestal. The ASE irradiation ends here and is followed by the exponentially increasing ramp.

Figure 1d,e show the line profiles of the electron number density at $y=5.5\, \mathrm {\mu m}$ and $x=0$ at the same time points as in Fig. 1c, respectively. At $t=-200\,\text{ps}$ the transverse profile remains intact in Fig. 1d and only the longitudinal compression is seen at $y=5\,\mathrm {\mu m}$ in Fig. 1e. Then both transverse and longitudinal ablation can be seen at $t=-150\,\text{ps}$ where the plasma expands from its initial position. From $t=-100\, \text{ps}$ to $t=-30\, \text{ps}$, we can clearly see that the electron density is compressed into the center of the nanowire. That is to say, the electron density distribution is imploded radially inwards following the ablation. The longitudinal ablation has the same characteristics as in the case of a plane target with an exponential pre-plasma profile. Hence, a 1D simulation along the longitudinal direction is not sufficient to reveal the difference between a nanowire and a plane target. The electron and ion temperature profiles after the ASE heating are also plotted in Fig. 1d,e on the right vertical axis. The ablated aluminum with density $n_i=n_\text{cr}$ is heated to around $T_i\sim 10\,\text{eV}$. The ion-ion mean free path is $\lambda _{ii} = 2.04\times 10^{13} T_i^2/(Z^4 n_i \ln \Lambda )\sim 0.02\,\text{nm}$ with the charge state, $Z=4$ (the Coulomb logarithm is found to be $\ln \Lambda \sim 2$). The mean free path is less than the nanowire diameter and the plasma is collisional. The electron and ion temperature in the ablated region is approximately equal, $T_e\sim T_i$.

**Figure 2:**
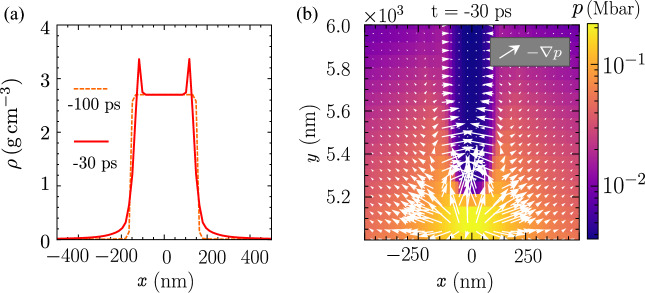
(**a**) The mass density transverse profile at $y=5.5 \, \mathrm {\mu m}$, and $t=-100,\,-30,\, \text{ps}$ under irradiation by the laser prepulse. (**b**) The pressure profile at $t=-30\, \text{ps}$ near the tip of the nanowire. The arrows indicate the specific force, $-\nabla p$ of the plasma.

Figure 2a shows the mass density compression begins at $t=-30\, \text{ps}$. The mass expands radially outward at a slower rate compared to the electron due to its higher inertia throughout the ASE pedestal irradiation. This explains why the mass density remains intact while the electron is already ablated at $t=-100\, \text{ps}$. Figure 2b shows the total plasma pressure and the specific force, $-\nabla p$ of the plasma near the tip at $t=-30\, \text{ps}$. The specific force represents the force of ablation pressure and implosion. The maximum plasma pressure is roughly $0.2\,\text{Mbar}$ at the tips, and the force generated by this pressure diverges outward in all directions. On the side surfaces of the nanowire, we can clearly distinguish the region of ablation indicated by arrows pointing away from the nanowire, and the area of implosion indicated by arrows pointing towards the nanowire. This provides the clear evidence of nanowire implosion by laser ASE pedestal.

**Figure 3:**
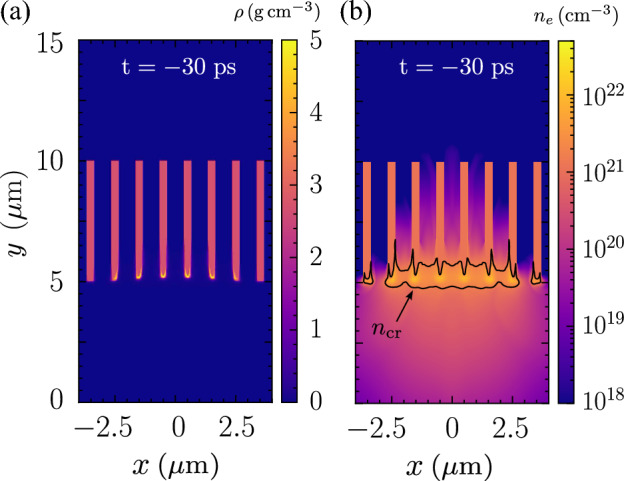
flash simulation of an array of nanowires. (**a**) The mass density, and (**b**) electron number density at $t=-30\, \text{ps}$. The critical density plasma blocks further laser penetration.

**Figure 4:**
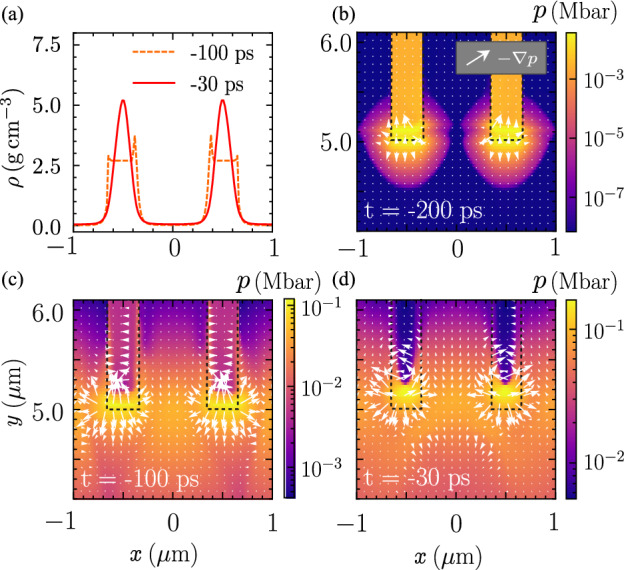
(**a**) The mass density profile at $y=5.2\,\mathrm {\mu m}$ for $t= \, -100 \, \text{ps},\, \text {and} -30\, \text{ps}$. The profiles only show two nanowires at the center of the array. The pressure profile at (**b**) $t=-200\, \text{ps}$, (**c**) $t=-100\, \text{ps}$, and (**d**) $t=-30\, \text{ps}$. The arrows indicate the direction of the specific force, $-\nabla p$.

### Nanowire array

To consider a more practical target configuration, we perform the same simulation using 8 nanowires with an interwire distance of $D=1\,\mathrm {\mu m}$. All simulation parameters are equivalent to the case of a single nanowire. Figure 3a shows an enhanced mass compression at $t=-30\, \text{ps}$ within the laser focusing spot. The tip exhibits a degradation with a length of $0.5\,\mathrm {\mu m}$. Only the nanowires at the center are compressed radially inwards, while the nanowires at the sides are pushed outwards. A near-homogeneous layer of plasma at critical density is formed in front of the nanowire as shown in Fig. 3b. The nanowire array is opaque to the laser pulse until the onset of relativistic transparency. In typical ion acceleration experiments, the electron density effects at the nanowire tips are similar to those on flat foil targets, and then continue with the new interactions relevant to the nanowire array, such as z-pinch and high-pressure generation^8,42^. This is only the case if the intensity of the main pulse exceeds the threshold of the relativistic transparency. Otherwise, there will no measurable differences as reported in Ref.^43^.

The transverse profile of the mass density of the two nanowires situated at the array’s center is illustrated in Fig. 4a. The nanowires are first compressed from the inner side at $t=-100\, \text{ps}$ and up to $1.8\times$ of their initial density at $t=-30\, \text{ps}$. The significant compression compared to a single nanowire at the same time point as shown in Fig. 2, is attributed to the ablation pressure from the surrounding nanowires. This is demonstrated in Fig. 4b–d, where the ablated plasma propagates from one nanowire to the next nanowire. The compression force among the nanowires, as depicted in Fig. 4c is directed towards the adjacent nanowires. The compression of the inner side is larger than the outer side due to the laser intensity difference in the focusing spot as the ablation pressure is intensity-dependent. The ablation pressure is written as $p_a[\text{Mbar}] = 8.6 \left( I_{14}/\lambda _{\text{L}}[\mathrm {\mu m}] \right) ^{2/3} \left( A/(2Z) \right) ^{1/3}$, where $I_{14}$ is laser intensity on target in the unit of $10^{14}\, \mathrm {W \, cm^{-2}}$, and *A* and *Z* are the mass number and the atomic number, respectively of the target materials^44,45^. As the irradiation continues, the ablation pressure from both sides of the neighboring nanowire increases, and the mass density is compressed radially inwards as shown in Fig. 4d.

**Figure 5:**
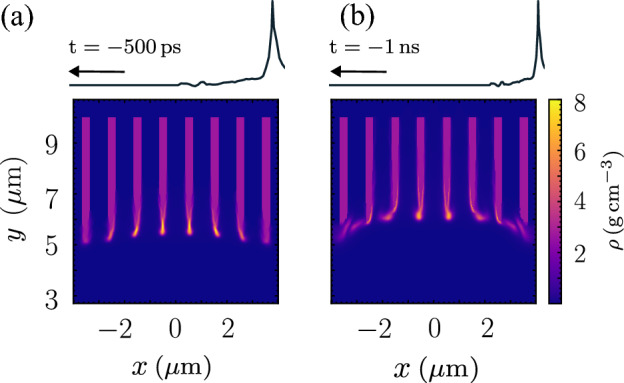
Mass density of an array of nanowire when the duration of the laser ASE pedestal is extended to (**a**) $-500 \, \text{ps}$, and (**b**) $-1 \, \text{ns}$.

## Discussion

Suppose the laser contrast of the same level is measurable up to $-500\, \text{ps}$ and $-1\, \text{ns}$ as shown in Fig. 5a,b respectively. A stronger mass compression due to the implosion remains on the nanowire around the centre of the focal spot. The nanowires near the edge of the focusing spot are bent outward by an uneven implosion due to the spatial difference in intensities. As a result, the array of nanowires resembles a crater drilled by the ASE. The longer the duration of the ASE, the deeper the crater formation, as depicted in Fig. 5b.

Implosions play a fundamental role in various applications, including plasma-based x-ray sources^46^ and the generation of ultrahigh magnetic fields^47^. In most cases, it is desirable to minimise its effects. Specifically, implosion results in target deformation that reduces the efficiency of laser-driven proton acceleration^43,48–50^. One possible approach to mitigate the implosion is to reduce the laser ASE pedestal energy, such as by using the frequency-doubled laser pulse. It should be noted that increasing the contrast ratio often leads to a decrease in the intensity of the main pulse.

**Figure 6:**
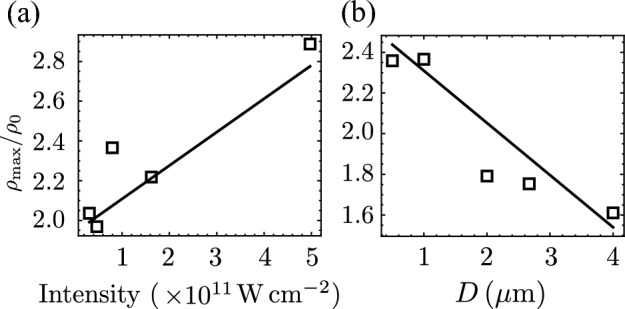
Scaling of the maximum mass compression of aluminum nanowire versus **a** different ASE intensities by changing the focal spot irradiated on nanowire array with spacing $D=1\,\mathrm {\mu m}$, and **b** nanowire with different spacing irradiated by ASE of intensity $10^{11}\, \mathrm {W \, cm^{-2}}$. The duration of the lase ASE pedestal is $250 \, \text{ps}$ for both cases. The solid lines are the linear fit of the data.

In Fig. 6, the correlation of the maximum mass compression of aluminum nanowire to the laser ASE intensities and spacing of nanowires in an array are presented. The $250 \, \text{ps}$ laser ASE of intensities in Fig. 6a were adjusted from $3.1\times 10^{10}\, \mathrm {W \, cm^{-2}}$ to $5.0 \times 10^{11}\, \mathrm {W \, cm^{-2}}$ with different focusing spots, $R_x=3.2, \, 2.6, \, 2.0, \, 1.4, \, \text {and} \, 0.8 \, \mathrm {\mu m}$. This scaling assumes that the laser contrast is fixed or cannot be further improved, and the intensities can only be reduced by changing the focal spot. The simulation results exhibit the increasing dependency of mass compression over the intensities. The RHD is unable to simulate the solid-to-plasma phase transition and therefore the immobile fluid approximation described in the “Methods” Section was used for the nanowire target. As a result, we would expect no compression at intensities near the LIB threshold. The interwire spacing considered in Fig. 6b were $D=0.5, \, 1.0, \, 2.0, \, 2.67, \, \text {and} \, 4.0 \, \mathrm {\mu m}$ and irradiated by $250 \, \text{ps}$ ASE of intensity $\sim 10^{11}\, \mathrm {W \, cm^{-2}}$. This scaling assumes that the laser intensity is fixed and the effects of implosion can be reduced via target fabrication. The compression is expected to resemble that of a flat foil for spacing smaller than the diameter of the nanowire. In contrast, at distances much larger than the nanowire diameter, the compression should saturate to that of a single nanowire.

Although the ablation pressure is weakly dependent on the target material, but the ablation or LIB threshold is material dependent. Therefore, choosing a material with a higher LIB threshold such as fused silica could help delay the ablation process. For example, in a long pulse regime the intensity of LIB threshold is, $I_0 \cong (\kappa t)^{1/2}\, \varepsilon _b n_a /(At)$, where $\kappa$ is the coefficient of thermal diffusion, $\varepsilon _b$ is the heat of evaporation per atom, *t* is the pulse duration, *A* is the absorption coefficient, and $n_a$ is the neutral atom density^33^. Then the intensity of LIB threshold for fused silica is calculated to be $I_0 \cong 8.2\times 10^{10} \, \mathrm {W \, cm^{-2}}$, one order larger than most metallic targets (taking $\kappa =0.0087\,\mathrm {cm^2 \, s^{-1}}$; $\varepsilon =3.7 \, \mathrm {eV/atom}$; $n_a=7\times 10^{22}\,\mathrm {cm^{-3}}$; $A\sim 3\times 10^{-3}$; and $t=250\,\text{ps}$). Materials with low absorptivity, such as transparent nanowire arrays^51,52^, could have a significant role in reducing the damage. In addition, target made of formvar has LIB threshold at $\sim 10^{11} \, \mathrm {W \, cm^{-2}}$ for $200\,\text{ps}$ ASE as described in Ref.^41^. Choosing the appropriate target material and wire spacing is by far the most direct and cost-effective way of reducing nanowire damage in high-intensity experiments.

The simulations do not take into account the support layer to avoid reflection effects of the laser rays. Therefore, this approximation is only applicable to sufficiently long or high aspect ratio nanowires to avoid interaction of the ablation plasma with the support layer. During the initial irradiation of a high aspect ratio nanowire, the laser at normal incidence heats both the substrate and the nanowire tip. Some laser rays are reflected and bounce in the gap between the nanowires in an array. These trapped rays heat the nanowire side surface and may results in the lateral expansion. Once the tip ablate and fills the gap within $\sim 50 \, \text{ps}$ as evidence in Fig. 4a, the critical plasma density prevents further laser heating of the substrate. At this time the implosion has not yet begin, and the freestanding nanowire approximation would be valid to scrutinize the effect at the tip. For a nanowire with a low aspect ratio, the effect of ray trapping is relatively weak. It can be reasonably anticipated that the plasma ablated from the support layer will have sufficient time to combine with the plasma at the tip to form a homogeneous pre-expanding plasma similar to a flat foil.

The 2D nanowire depicted in Fig. 1 corresponds to a slab with infinite extension in the $z$-direction in three-dimensional space. The mass density is written as $\rho = m/(2r_0 \times y \times z)$, where *m* is the mass of the target, $2r_0$ is the nanowire diameter, and $z = \pm \infty$. For a finite compression length along the $y-$axis, the compression factor is $\rho /\rho _0 = r_0/r$. If we consider the nanowire as a cylindrical rod in three-dimensional space, we can express its volume as $V=\pi r_0^2 y$. The compression factor can be determined as $\rho /\rho _0 = (r_0/r)^2$. Therefore, the lateral compression of the nanowire is expected to increase by $(n-1)$, where *n* is the number of dimensions. A 1D simulation would show no compression, whereas in 3D a maximum compression by a factor of $2.9^2$ can be estimated following the result in Fig. 6a for an ASE intensity of $5 \times 10^{11} \, \mathrm {W \, cm^{-2}}$.

Materials with a higher atomic number will exhibit a higher plasma pressure, which is given by $P = (Z + 1) n_ekT$, where *Z* is the atomic number, *k* is the Boltzmann constant, and *T* is the temperature of the target by assuming thermal equilibrium between electron and ion. However, the ablation pressure has a weak dependence on the material. At the same laser ASE intensity, the ablation pressure would not be strong enough to overcome the plasma pressure of the gold target. Therefore, we expect implosion to be less pronounced for high-Z materials.

Several challenges remain in the hydrodynamic simulations of laser prepulse interaction with nanostructured targets, specifically, the need to address the mitigation of free plasma expansion in RHD simulation. The present method for handling the solid at room temperature only gives an approximation when modelling the target strength. To achieve a more accurate description of laser prepulse nanowire interaction, simulations of molecular dynamics and phase change when heated by the laser prior to ablation are necessary. This will also prevent any negative pressure issues in the EOS.

The lightning rod effect^53–55^ is expected to enhance the field at the tip. The effective intensity at the round tip of the nanowires is written as, $I_\text{eff} = I_\text{ASE}\left[ (L_\bot ^\text{surf})^2 \sin ^2\theta + \cos ^2\theta \right]$, where $L_\bot ^\text{surf}$ is the local field correction coefficient and $\theta$ is the laser incidence angle. The local field correction factor depends weakly on material permittivity for high aspect ratio nanowires and decreases with increasing nanowire radius. An estimated local field correction factor for aspect ratios $>30$ is in the range of 1 to 10. For small incident angle, $\theta \sim 10^\circ$ we have $I_\text{eff} \sim 4 I_\text{ASE}$. The effective intensities at $t = -30\,\text{ps}$ is estimated to up to $\sim 10^{12}\,\mathrm {W\, cm^{-2}}$. In addition, the photoelectrons emission from the target via a surface-plasmon-assisted multiphoton photoelectric process could happen at much lower intensities than the lightning rod effect, typically of the order of $10^{9} \, \mathrm {W \, cm^{-2}}$^56^. These effects would cause the LIB to begin at a much earlier time than expected from the BSI threshold, where a similar phenomenon has been reported in Ref.^57^. These processes are not able to be simulated with flash but one could expect an increase in electron density and hence the ablation pressure. This will result in a more pronounced effect of ablation and implosion.

## Conclusion

In conclusion, we have observed the implosion of nanowires after irradiation with a $250\,\text{ps}$ laser ASE of a $1\,\text{PW}$ laser pulse. The implosion of the nanowires is caused by a rocket-like propulsion of the ablated plasma. This leads to mass compression before the arrival of the main pulse. The mass compression is more significant when a denser array of nanowires is used. This is due to ablation pressure from neighbouring nanowires, which adds to the compression force. The deformation scales with ASE duration, intensity and wire spacing. A key finding was that the ASE contrast at the level of $10^{12-13}$ of the PW class laser is insufficient to avoid damage to most metallic nanowires. Considering the LIB threshold and the field enhancement as discussed, we estimate the ASE contrast at the level of $\gtrsim 10^{15}$ is required for the experiment performed with the main laser pulse of peak intensity $\gtrsim 10^{22}\,\mathrm {W\, cm^{-2}}$. Our results also demonstrate the importance of studying the material properties in the search for higher LIB thresholds.

## Methods

### Simulation setup and parameters

flash code consists of standard hydrodynamic solvers extended with multi-temperature treatment for plasma in laser-driven high-energy-density physics experiments. The ion, electron, and radiation are assumed not in thermal equilibrium (3T). Energy exchange between electrons and ions, electron thermal conductivity, and laser energy deposition via inverse bremsstrahlung are included. Material properties such as the equation of state (EOS), average ionization state, and opacities are obtained from ionmix4 tabulated data^58^. Radiation diffusion is incorporated using the multi-group diffusion (MGD) theory^40^.

The simulation setup is modified from the laserslab test problem in flash. The simulation domain $x\times y$ has the size of $4 \times 15 \, \mathrm {\mu m^2}$ in a Cartesian coordinate system. We have used AMR with $4-5$ levels of refinements over the mass density and electron temperature. The highest resolution is $15.6 \times 58.6 \,\text{nm}$. The simulation time-step is between $1\times 10^{-16}-3\times 10^{-9} \, \text{s}$ with Courant-Fiedrichs-Lewy (CFL) number of 0.4. The physical parameters in the simulation are in the CGS unit.

### Radiation transport and conductivity

The total radiation energy density, emission, and absorption terms in the MGD solver are divided into 6 groups with energies boundaries of $10^{-1}, 10^0, 10^1, 10^2, 10^3,10^4, 10^5$ with the harmonic limiter. The boundary condition for radiation diffusion is set to vacuum. We used the Spitzer model for heat exchange and thermal conductivity with an electron conductivity flux-limiter coefficient of 0.06. The temperature gradient is assumed to be zero at the simulation boundaries.

### Nanowire target

**Figure 7:**
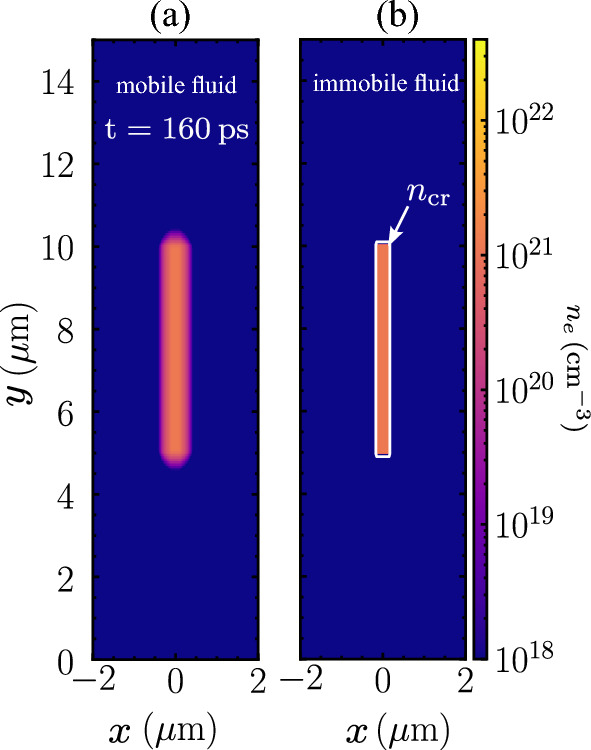
The condition of the nanowire without laser irradiation when (**a**) the fluid is mobile, and (**b**) the fluid is immobile after $160\,\text{ps}$ of runtime. The fluid in (**a**) undergoes free expansion even if no laser irradiation. The white line in (**b**) indicates the critical density contour. The fluid motion in (**b**) is set to mobile if the target temperature reaches $T=933 \, \text{K}$.

Aluminum nanowire of $5 \, \mathrm {\mu m}$ length and $300 \, \text{nm}$ diameter is considered. The choosen nanowire diameter and length are within the range of optimal laser absorption and penetration depth reported in Ref.^3^. The aluminum has the mass density, $\rho =2.7\,\mathrm {g \, cm^{-3}}$ at room temperature. flash code does not implement additional physics related to the solid strength and phase transition. The solid will undergo free expansion even if there is no laser irradiation as shown in Fig. 7a. To handle the room temperature solid, we adopted a method to set the fluid motion immobile at room temperature until it is heated by the laser to the melting temperature at $T=933\,\text{K}$. Figure 7b shows that the solid does not expand after employing the aforementioned method after $160\,\text{ps}$ without laser irradiation. The supporting substrate is not included in the simulation to exclude the effects of laser ray reflection. We note that the lateral periodic boundary condition does not work in representing the nanowire outside the boundary, because the laser is modeled with a ray tracing algorithm instead of a plane wave as in particle-in-cell simulations.

### Laser

The laser energy absorption proceeds through inverse bremsstrahlung and the laser propagation is modeled by a ray-tracing algorithm using 2000 rays with second-order correction. The laser intensity has 1-dimensional Gaussian spatial profile, $I(x)=I \exp (x^2/R_x^2)$, where $R_x=w_0/\sqrt{2}$ and $w_0$ is the laser waist radius. The laser wavelength is $\lambda _\text{L}=820 \, \text{nm}$, and focused into $R_x=2\,\mathrm {\mu m}$. Here, *I* is the intensities set by the laser temporal profile. The laser temporal profile in Fig. 1b is obtained from the measured $1\,\text{PW}$ contrast at ELI-NP^38,39^. A total of 43 points from $-250\,\text{ps}$ to $30\,\text{ps}$ are sampled from the measured profile, with 35 points between $-250\,\text{ps}$ and $-10\,\text{ps}$. This pulse maintained its ASE contrast level at $10^{11}$ for $230 \, \text{ps}$ and ramp-up exponentially for $20 \, \text{ps}$ before the peak laser intensity at $t=0$ as shown in Fig. 1b. With the main pulse at $I_0\sim 10^{22} \, \mathrm {W \, cm^{-2}}$ or $a_0=70$, the ASE pedestal intensity is estimated to be $\sim 10^{11} \, \mathrm {W \, cm^{-2}}$. Here, $a_0=eE_0/(mc\omega _\text{L})$ is the normalized laser amplitude, where $E_0$ is the peak laser field strength, $\omega _\text{L}=2\pi /\lambda _\text{L}$ is laser central frequency, and *e* and *m* being the charge and mass of electron.

### Ionization

**Figure 8:**
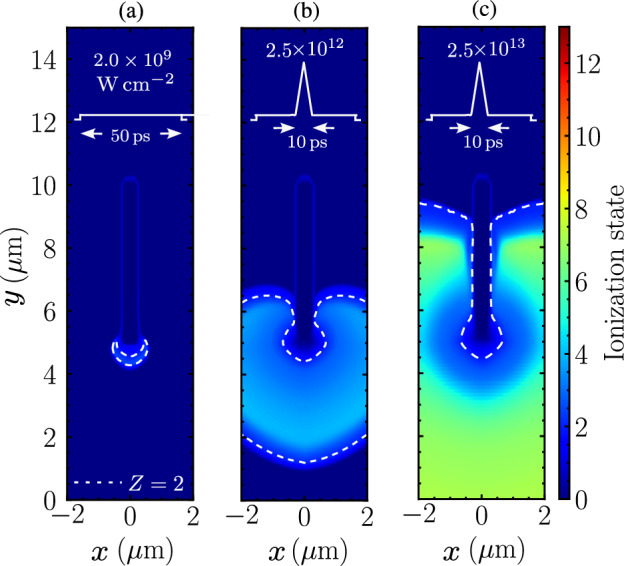
Comparision of nanowire ablation when irradiated by laser with (**a**) $50\,\text{ps}$ ASE of intensity $2 \times 10^{11} \, \mathrm {W \, cm^{-2}}$ , and $50\,\text{ps}$ ASE consisting of one $10\,\text{ps}$ pulse with peak intensity (**b**) $2.5 \times 10^{12} \, \mathrm {W \, cm^{-2}}$, and (**c**) $2.5 \times 10^{13} \, \mathrm {W \, cm^{-2}}$.

The collisional ionization takes place when the target is heated by the laser ASE pedestal. The electron densities are determined by the average ionization state from the tabulated data as the function of the local mass density and temperature. The aluminum has already been one time ionized by $50 \, \text{ps}$ ASE pedestal via the collisional ionization as shown in Fig. 8a. The presence of short prepusles with higher intensities along the ASE pedestal will increase the ionization state. The charge state of the aluminum ion when irradiated by $10 \, \text{ps}$ prepulse with peak intensity $2.5\times 10^{12} \, \mathrm {W \, cm^{-2}}$ is up to $Z=4$ as presented in Fig. 8b. Keeping the same pulse duration and increase the peak intensity by one order of magnitude, the ion charge is now up to $Z=8$ as shown in Fig. 8c. The electron density during the initial ASE irradiation has already resulted in $n_e=1.2 \times 10^{23}\,\mathrm {cm^{-3}}>> n_\text{cr}$. Therefore, the critical density shielding prevents the nanowire from further ionization, and the electron density is lower at the tip than the surrounding.

At higher laser intensities, the strong field ionization such as multiphoton ionization, tunneling ionization, and barrier suppression ionization (BSI) can take place^59–61^. The threshold field strength of the BSI is expressed as the appearance intensity, $I_\text{app}$^62^. This appearance intensity is written as

1 $$\begin{aligned} I_\text{app} [\mathrm {W \, cm^{-2}}] \approx 4 \times 10^9 \frac{E_\text{ip}[\text{eV}]^4}{Z^2} \end{aligned}$$,

where $E_\text{ip}$ being the ionization potential of the atom or ion with charge $Z-1$. The onset of the LIB threshold is often estimated using the appearance intensity. The appearance intensity for Al$^{+}$ is $5.1\times 10^{12} \, \mathrm {W \, cm^{-2}}$ ($E_\text{ip}=5.986\,\text{eV}$^63^). Further ionization to Al$^{2+}$ requires intensity $1.26\times 10^{14} \, \mathrm {W \, cm^{-2}}$ ($E_\text{ip}=18.83\,\text{eV}$).

However, the aluminum is ionized at least twice by collisional ionization. Therefore, ionization to the next higher level via BSI requires even higher intensities. One can assume that the strong field ionization would not play a dominant role during the ASE irradiation. The simulation stops at $t=-10\,\text{ps}$ before the main pulse, as the hydrodynamic simulation is not capable of describing collisionless absorption.

## Data Availability

The datasets used and/or analyzed during the current study are available from the corresponding author on reasonable request.
